# Anaesthesia and orphan disease: management of a case of Nicolaides-Baraitser syndrome undergoing cleft palate surgery

**DOI:** 10.1186/s12871-021-01380-z

**Published:** 2021-05-26

**Authors:** Marie Goehring, Suma Choorapoikayil, Kai Zacharowski, Leila Messroghli

**Affiliations:** Department of Anaesthesiology, Intensive Care Medicine and Pain Therapy, University Hospital Frankfurt, Goethe University Frankfurt, Theodor-Stern-Kai 7, 60590 Frankfurt am Main, Germany

**Keywords:** Case report, Nicolaides-Baraitser syndrome, Surgery, Anaesthesia

## Abstract

**Background:**

Nicolaides-Baraitser syndrome (NCBRS) is a rare disease caused by mutations in the SMRCA2 gene, which affects chromatin remodelling and leads to a wide range of symptoms including microcephaly, distinct facial features, recurrent seizures, and severe mental retardation. Until now, less than 100 cases have been reported.

**Case presentation:**

A 22-month old male infant with NCBRS underwent elective cleft palate surgery. The anaesthetists were challenged by the physiological condition of the patient: narrow face, very small mouth, mild tachypnea, slight sternal retractions, physical signs of partial monosomy 9p, and plagiocephalus, midface hypoplasia, V-shaped cleft palate, enhanced muscular hypotension, dysplastic kidneys (bilateral, estimated GFR: approx. 40 ml/m2), nocturnal oxygen demand, and combined apnea. In addition, little information was available about interaction of the NCBRS displayed by the patient and anaesthesia medications.

**Conclusions:**

The cleft palate was successfully closed using the bridge flap technique. Overall, we recommend to perform a trial video assisted laryngoscopy in the setting of spontaneous breathing with deep inhalative anaesthesia before administration of muscle relaxation to detect any airway difficulties while remaining spontaneoues breathing and protective reflexes.

## Background

Nicolaides-Baraitser syndrome (NCBRS) is caused by mutations in the *SMRCA2* gene, which affects chromatin remodelling and leads to a wide range of symptoms including sparse scalp hair, microcephaly, distinct facial features, short stature, recurrent seizures, and severe mental retardation [1–6]. Until now, less than 100 cases have been reported and information to perform anaesthesia in these patients are missing. This case report is about a 22-month old male infant with NCBRS who underwent elective cleft palate surgery in July 2020. Informed consent for publication of this case report was obtained from the parents.

## Case presentation

The patient weighed 8 kg and was 77 cm tall. During the pre-anaesthetic evaluation, the patient presented a good nutritional state, with a slightly short stature, narrow face, very small mouth, and delayed development (status of a 6 month). In addition, the patient showed a mild tachypnea, slight sternal retractions, physical signs of partial monosomy 9p, and plagiocephalus. No signs of stridor was observed. The medical history recorded a midface hypoplasia, V-shaped cleft palate, enhanced muscular hypotension, dysplastic kidneys (bilateral, estimated GFR: approx. 40 ml/m^2^), nocturnal oxygen demand, and combined apnea. Transthoracic echocardiography performed six months before the surgery revealed normal sized and well contractile ventricles. Colour and pulsed-wave Doppler revealed frail aortic and pulmonary valves with normal vessels including the pulmonary artery bifurcation and a normal flow profile of the aortic arch and aortic isthmus. Additionally, the patient shows frail atrioventricular valves, normal conditions in the area of both atria including the supplying pulmonary, hepatic and vena cava veins. An atrial septal defect of secondary type with a slight left-right shunt was found in the atrial septum. No pericardial or pleural effusion was detected. The assessment of atrium-septum-defect (ASD II) revealed no evidence of hemodynamic relevance. The mother is known to have a clinically evaluated mild form of von Willebrand syndrome and a factor V mutation. Tests revealed, that both diseases were not inherited to the infant.

The patient was prepared for surgery following routine protocols. The temperature in the operating room was increased to 24 °C. An air blow blanket “Mistral AIR” set to 38 °C was used additionally. As a premedication the patient received 2.5 mg midazolam as suppository and was fully relaxed after 10 min. Nose drops (xylometazoline (0.25 mg/ml)) were administered to ease nasal breathing. General anaesthesia was initiated by inhalative application of sevofluran. We started pre-oxygenation with Fi0_2_ 1.0 until etO_2_ > 80% and sevoflurane 6 Vol%. Mask ventilation under pressure support ventilation went smoothly and without any problems. Patients with NCBRS often display craniofacial dysmorphic features. Since our patient had a very flat face, and possessed a very small mouth with small lips we expected possible airway difficulties. Based on this we performed a trial laryngoscopy in the setting of spontaneous breathing with deep inhalative anaesthesia. We used a video laryngoscope (C-Mac©) for laryngoscopy due to the expected airway difficulties. However, no complications occurred and direct laryngoscopy was easily manageable and described as ‘Cormack and Lehane system grade II’ (CL II) we deepened the anaesthesia with fentanyl (15 mcg) and muscle relaxation was established with rocuronium (5 mg). Afterwards an atraumatic and problem-free intubation with C-Mac© under vision, CL II (conventional) was performed. We noticed bilateral breathing sounds, CO_2_ measurements were positive, no damage to teeth and soft tissue occurred, tube ID size 4.0 was used, cuff pressure 5 cmH_2_O via continuous measurement was established and the breathing tube was fixated at 11 cm tooth rack. Eye protection according to standard procedures was established. Pupils were both narrow and isocor. A gastric tube was inserted to relief gastric pressure and to ensure elimination of air. Then, several monitoring tools were established: rectal temperature probe, non-invasive blood pressure cuff.

The uncomplicated cleft palate was closed using the bridge flap technique with repositioning of the palatal elevation muscles. Intraoperatively, there was a non-specific slight bleeding. Since Willebrand syndrome was excluded we hypothesize that the slight bleeding occurred because patients undergoing cleft palate surgery receive administration of local anaesthesia in the upper jaw which leads to loosening of the tissue and fluid/blood leakage. There was also an ear-nose-throat (ENT) / paedaudiological control with paracentesis in case of seromucotympanum and insertion of tympanic drainage type “Goode”. Furthermore, a small upper lip ligament and a small tongue ligament were cut with scissors.

Pain relief medication (titrated fentanyl (initially 15 mcg, repeated by 35 mcg and 15 mcg) intraoperatively and metamizole (dipyrone; 120 mg) as postoperative pain killer) as well as clonidine (10 mcg) as co-analgesic drug were applied. Approximately 40 min after the initial administration of rocuronium, sevoflurane and fentanyl, the child showed triggering, so that ventilation was switched to spontaneous pressure controlled ventilation. Similar to other maxilla facial surgical procedures complete return of the protective reflexes could only be recognized at the end of the anaesthesia. At the nearly end of the surgery the supply of anaesthetic gas was slowly reduced and stopped during the last suture and process of complete washing out of the inhalative sedation was initiated. The time from wound closing until extubation was 12 min. After 10 min of surveillance in the operating room, the patient was transferred to the intensive care unit for further observation. However, there was no urgent need for intensive care treatment. The postoperative course was unremarkable without a need for additional oxygen supply. The patient stayed under intensive medical supervision for one night and was then referred to the general ward. The postoperative wound healing was regular. Discharge from hospital took place with irritation-free wound conditions and intraorally remaining absorbable suture material, continued feeding via nasogastric tube and continued nocturnal oxygen demand.

## Discussion and conclusions

NCBRS is a rare disease and information to treat these patients are missing, particularly to anesthetize infants. In this case report, we provide information about our approach to perform anaesthesia in a 22-month old infant with NCBRS. Since NCBRS is often associated with midface abnormalities resulting in difficult airway, we recommend using a video assisted laryngoscopy to reduce risks associated with this physical appearance. A first tentative laryngoscopy during spontaneous breathing with deep inhalation anaesthesia provided information about potential difficulties, which might occur during the intubation process. It is noteworthy to mention, that volatile anaesthetic agents are related to cause mild muscle relaxation. This could lead to complications in case of patients with impaired muscle tonus especially the respiratory muscles and diaphragm could be affected. However, patients remain spontaneous breathing and protective reflexes during induction with volatile anaesthesia. In addition, to avoid any long-term hospital related anxiety by establishing intravenous lines in paediatric patients we mostly administer volatile anaesthesia for initial induction in our hospital. This approach also allowed us to perform a trial video assisted laryngoscopy which is more complicated and might even not be possible with intravenous anaesthetic agents. No information was existing about the interaction of NCBRS and anaesthesia pharmacology, our drugs chose has been made on our practical experience. Furthermore, the dosage of all medication was adjusted according to the reduced elimination rate of the kidney. We used sevoflurane and fentanyl without any clinical pharmacology modification or complication. Another case of NCBRS anaesthesia has been published since ours occurred. The authors managed differently the airwaves and did not report any complication [7]. Finally, we provide information to successfully anesthetize an infant with distinct facial appearance.

## Data Availability

Data used during the current study is available from the corresponding author on reasonable request.
